# Genome-Wide Characterization of bHLH Genes in Grape and Analysis of their Potential Relevance to Abiotic Stress Tolerance and Secondary Metabolite Biosynthesis

**DOI:** 10.3389/fpls.2018.00064

**Published:** 2018-02-01

**Authors:** Pengfei Wang, Ling Su, Huanhuan Gao, Xilong Jiang, Xinying Wu, Yi Li, Qianqian Zhang, Yongmei Wang, Fengshan Ren

**Affiliations:** Shandong Academy of Grape, Jinan, China

**Keywords:** grape, bHLH transcription factor, abiotic tolerance, flavonol, anthocyanins, E-box, G-box

## Abstract

Basic helix-loop-helix (bHLH) transcription factors are involved in many abiotic stress responses as well as flavonol and anthocyanin biosynthesis. In grapes (*Vitis vinifera* L.), flavonols including anthocyanins and condensed tannins are most abundant in the skins of the berries. Flavonols are important phytochemicals for viticulture and enology, but grape bHLH genes have rarely been examined. We identified 94 grape bHLH genes in a genome-wide analysis and performed Nr and GO function analyses for these genes. Phylogenetic analyses placed the genes into 15 clades, with some remaining orphans. 41 duplicate gene pairs were found in the grape bHLH gene family, and all of these duplicate gene pairs underwent purifying selection. Nine triplicate gene groups were found in the grape bHLH gene family and all of these triplicate gene groups underwent purifying selection. Twenty-two grape bHLH genes could be induced by PEG treatment and 17 grape bHLH genes could be induced by cold stress treatment including a homologous form of MYC2, VvbHLH007. Based on the GO or Nr function annotations, we found three other genes that are potentially related to anthocyanin or flavonol biosynthesis: VvbHLH003, VvbHLH007, and VvbHLH010. We also performed a cis-acting regulatory element analysis on some genes involved in flavonoid or anthocyanin biosynthesis and our results showed that most of these gene promoters contained G-box or E-box elements that could be recognized by bHLH family members.

## Introduction

The bHLH protein is a kind of transcription factor, and it has DNA binding and dimerization capabilities as bHLH domain existed. bHLH domain contains approximately 60 amino acids with two functionally distinct regions of the HLH region and the basic region (Murre et al., 1989; Ledent and Vervoort, 2001). The HLH region contains two amphipathic α helices separated by a loop region of variable length which could act as a dimerization domain that promote protein-protein interactions and form homo-dimers or hetero-dimers (Massari and Murre, 2000). The basic region contains approximately 17 amino acids, and it is located at the N-terminus of the bHLH domain which allows bHLH transcription factors to bind to a consensus E-box (5′-CANNTG-3′) or G-box (5′-CACGTG-3′) cis elements to regulate gene expression (Atchley et al., 1999; Massari and Murre, 2000; Toledo-Ortiz et al., 2003; Li et al., 2006).

bHLH transcription factor family is very large in eukaryotes (Ledent and Vervoort, 2001; Toledo-Ortiz et al., 2003). There are 162 bHLH genes identified in Arabidopsis (Bailey et al., 2003), 167 bHLH genes in rice (Li et al., 2006), and 152 bHLH genes in tomato (Wang et al., 2015).

bHLH transcription factors could act as transcriptional activators or repressors and play important roles in metabolic and developmental processes (Feller et al., 2011). Most bHLH proteins identified have been functionally characterized in Arabidopsis. For example, PIF3 is a direct phytochrome reaction partner in the signaling network of photoreceptors, and it is involved in controlling the expression of light-regulated genes (Toledo-Ortiz et al., 2003). PIF4 plays a major role in growth regulation by integrating with multiple signals (Choi and Oh, 2016). bHLH transcript factor NAI1 is involved in the formation of endoplasmic reticulum bodies. RHD6 and RSL1 are involved in the formation of root hairs while LHW is involved in root development. PRE1, PRE2, PRE3, PRE4, and PRE5 are involved in gibberellin signaling transduction in plants (Feller et al., 2011).

Some bHLH family members have also been reported to be related to responses to abiotic stresses such as cold, drought, and salt stress. the tomato bHLH transcription factor MYC-type ICE, SlICE1a, could confer cold, osmotic, and salt tolerance of plants (Feng et al., 2013). A stress-responsive gene, bHLH transcript factor RsICE1 from *Raphanus sativus* could increase the cold tolerance of genetically modified rice (Man et al., 2017). bHLH transcript factor ICE1 could increase cold tolerance of *Pyrus ussuriensis* by enhancing PuDREBa transcriptional levels through interacting with PuHHP1 (Huang et al., 2015). Arabidopsis bHLH92 could function in plant's responses to osmotic stresses (Jiang et al., 2009). *Eleusine coracana* bHLH57 are related to drought, salt, and oxidative tolerance while bHLH122 plays an important role in drought and osmotic resistance in Arabidopsis (Liu et al., 2014). Arabidopsis bHLH112 was also reported to regulate the expression of genes that involved in abiotic stress tolerance (Liu et al., 2015). In addition to participate in adversity responses, bHLH genes in plants were also found to be involved the biosynthesis pathways of anthocyanins (Mol et al., 1998; Nesi et al., 2000; Winkel-Shirley, 2001) and flavonols (Walker et al., 1999; Nesi et al., 2000; Baudry et al., 2004).

Grapes (*Vitis vinifera L*.) are one of the most widely cultivated fruit crops in the world, and they are of great economic importance. However, grape production is often severely limited by various abiotic stresses such as low temperature and winter droughts. In northern China of the main grape-producing areas, there have almost no grape cultivars survive under natural conditions of low temperatures and low air humidity in the winter because of the continental climate (Huang, 1980). Since the bHLH family has been proven to be associated with abiotic stress tolerance in many species, we want to identify bHLH transcription factors in grapes to analysis their potential relevance to cold and drought tolerance.

Here, a total of 94 grape bHLH genes were identified in grape genome, and phylogenetic analyses were carried out to evaluate the relationships among these genes. The expression patterns of 94 grape bHLH genes in various tissues under cold and drought stress conditions were analyzed. Three grape bHLH genes that may have relevance to anthocyanin and flavonol biosynthesis were identified. Our purpose of this study was to find more candidate genes that may have relevance to cold and drought stresses tolerance, and to improve the current understanding of cold and drought tolerant mechanisms in *V. vinifera*. We also aimed to identify candidate genes that may participate in anthocyanin and flavonol biosynthesis for further breeding and cultivation of the new grape varieties.

## Materials and methods

### Data collection and identification of grape bHLH genes

The whole grape genome was obtained from the newest foxtail millet genome database (version v2.0; http://genomes.cribi.unipd.it/DATA/V2/). The conserved bHLH domain sequence (PF00010; http://pfam.xfam.org/) was used as a query to identify all possible bHLH protein candidates in the grape genome database using BLASTP (*E* < 0.001). SMART online software (http://smart.embl-heidelberg.de/) was used to identify integrated bHLH domains in putative grape bHLH proteins. The date of the query was December 2016. The bHLH genes identified were named according to their order in the *V. vinifera* genomic sequence.

### Protein and GO annotation

Nr annotations were based on the NCBI BLASTP and Nr databases (https://blast.ncbi.nlm.nih.gov/Blast.cgi?PROGRAM=blastp&PAGE_TYPE=BlastSearch&LINK_LOC=blasthome). GO function annotations were based on the GO database (http://geneontology.org/page/go-database).

### Phylogenetic analysis of grape bHLH members

We performed a grape bHLH protein sequence alignment using Clustal Omega online software (http://www.ebi.ac.uk/Tools/msa/clustalo), and constructed Neighbor-Joining (NJ) trees using MEGA 6.0 with the aligned grape bHLH protein sequences. One-thousand bootstrap samples were generated to support the calculated relationships (Wang et al., 2016).

### Gene duplication and triplication analyses

Grape bHLH duplicate gene pairs and triplicate gene groups were identified with two standards. Protein pairs with ≥30% identity, covering ≥70% of the protein length fell within the low-stringency standard. Protein pairs with ≥50% identity, covering ≥90% of the protein length were included in the high-stringency standard (Rizzon et al., 2006; Sun et al., 2015).

### Conserved motif analysis of grape bHLH protein sequences

We analyzed conserved motifs in grape bHLH protein sequences using the Multiple Em for Motif Elicitation (MEME) suite 4.11.1 software (http://meme-suite.org/tools/meme) (Bailey et al., 2006) with the following parameter settings: output motifs, 20; minimum motif width, 6; and maximum motif width, 300 (Wang et al., 2016).

### Selective pressure analysis

The non-synonymous/synonymous (Ka/Ks; ω) value of duplicate gene pairs or triplicate gene groups (between any two genes in one triplicate gene groups) were calculated using PAL2NAL (http://www.bork.embl.de/pal2nal/; Suyama et al., 2006; Yadav et al., 2016).

### Codon usage bias analysis

Codon bias refers to the unequal use of synonymous codons for an amino acid (Hershberg and Petrov, 2008; Larracuente et al., 2008; Plotkin and Kudla, 2011; Guo et al., 2017). Coding sequences of 94 bHLH genes were used to calculate the frequency of optimal codons (FOP), GC content, GC content at the 3rd site of the synonymous codon (GC3s content), relative synonymous codon usage (RSCU), codon adaptation index (CAI), and codon bias index (CBI) with the online software CodonW 1.4 (http://codonw.sourceforge.net).

A plot of the effective number of codons (ENC) vs. GC3s (also called an ENC plot) is a strategic investigation into patterns of synonymous codon usage, providing a visual display of the main features of codon usage patterns for a number of genes. The effective number of codon values are always within the range of 20 (only one codon effectively used for each amino acid) to 61 (codons used randomly). The expected ENC values were calculated as follows (Wright, 1990):

(1) $$\begin{array}{c} {\text{ENC}}_{\text{exp}}=2+\text{S}+(29/({\text{S}}^{2}+(1-{\text{S}}^{2}))) \end{array}$$

Where, S is the frequency of G + C (i.e., GC3s).

### Promoter cis-acting regulatory element analysis

Plantcare (http://bioinformatics.psb.ugent.be/webtools/plantcare/html/) was used to analyze the cis-acting regulatory elements of bHLH genes (Wang et al., 2016).

### *In silico* gene expression analysis

The microarray expression profiles of 49 grape samples (GSE36128) (Fasoli et al., 2012), PEG (simulated drought), cold stress library, control library (GSE31594; https://www.ncbi.nlm.nih.gov/geo/query/acc.cgi?acc=GSE31594), and CBF over-expression transgenic grape library (GSE29948; https://www.ncbi.nlm.nih.gov/geo/query/acc.cgi?acc=GSE29948) were retrieved from the Plexdb (http://www.plexdb.org/) and GEO databases (https://www.ncbi.nlm.nih.gov/gds/). A heatmap was generated using MultiExperiment Viewer (MeV) v4.0.

## Results

### Identification, phylogenetic analysis, and annotation of grape BHLH genes

We identified 94 bHLH genes in *Vitis vinifera*. They were named from VvbHLH001 to VvbHLH094 according to their order in the *V. vinifera* genomic sequence. All bHLHs were annotated based on the best-hit proteins in the Nr database (Table S1). Our Nr-annotation showed that the grape bHLH family contained some homologous bHLH genes that had been previously reported in Arabidopsis (Feller et al., 2011). However, the annotation did not contain the homologs of FIT (involved in the regulation of iron uptake), AIB (involved in ABA signaling), RHD6 (involved in root hair formation), RSL1, RSL4, RSL3, RSL2, RSL5 (involved in root hair development), PRE1, PRE2, PRE3, PRE4, PRE5 (involved in gibberellin signaling), SlSTYLE2.1 (involved in cell elongation in developing styles), KDR (involved in light signal transduction), and some orphans identified in Arabidopsis (Feller et al., 2011).

**Figure 1:**
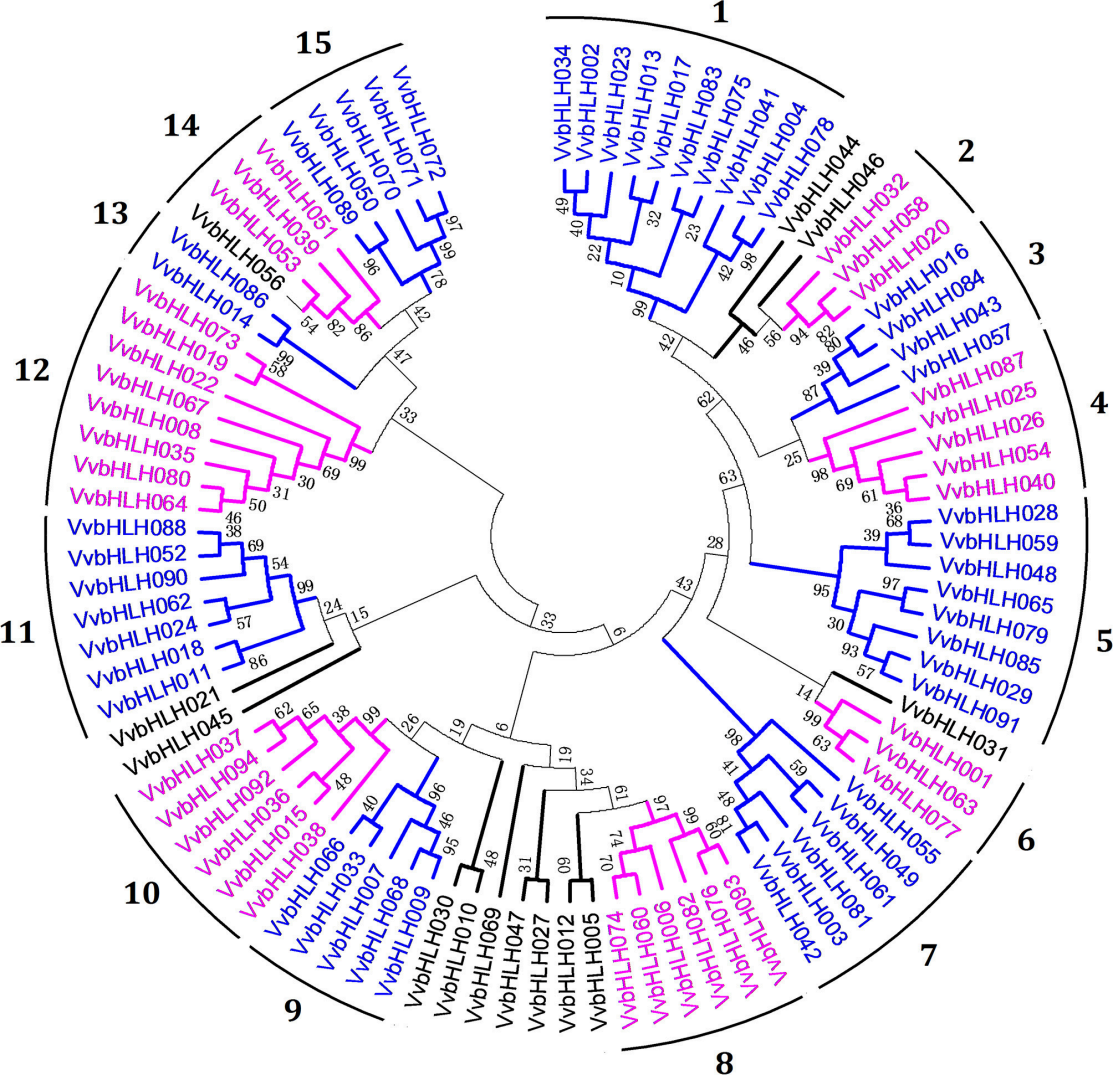
Neighbor-joining phylogenic analysis of grape bHLH family.

We used the full-length amino acid sequences of the 94 grape bHLHs for a phylogenetic analysis, in which subgroups with relatively high bootstrap support (≥70) were identified. The phylogenetic tree revealed 15 clades (groups 1–15) in the group bHLH family and 12 orphans (Figure 1). A phylogenetic tree with Arabidopsis showed that 17 grape bHLHs were tightly grouped with the AtbHLHs (bootstrap support ≥70) (Figure S1). They may be orthologous to the 17 AtbHLHs and have similar functions.

GO functional annotation analyses were performed to survey the function of all grape bHLH transcription factors (Table S2). All grape bHLH transcription factors could be divided into three main terms: cellular component, molecular function, and biological process. Our results indicated that many grape bHLHs may be involved in the regulation of transcription, as well as many biological processes such as abiotic stress tolerance (Abe et al., 2003; Lee et al., 2005), organ development (Feller et al., 2011), anthocyanin biosynthesis (Mol et al., 1998; Nesi et al., 2000; Winkel-Shirley, 2001), and flavonol biosynthesis (Walker et al., 1999; Nesi et al., 2000; Baudry et al., 2004).

Our GO functional annotation showed that many grape bHLHs may respond to stimuli, including 10 different types of biotic stressors and 25 abiotic stressors (Table S2) such as response to cold, drought, UV-B, fungus, and bacteria. Many grape bHLHs were involved in secondary metabolite biosynthesis including anthocyanin-containing compound biosynthetic processes, xylan biosynthetic processes, and fatty acid beta-oxidation (Table S2). GO functional annotation also showed that grape bHLHs may be involved in the development of 28 types of tissues or cells (Table S2) and 10 types of cellular components (Table S2).

### Structure analysis

Structure analyses of all of grape bHLH genes revealed that the number of exons varies from one to 11. Additionally, VvbHLH091, VvbHLH009, VvbHLH033, VvbHLH066, and VvbHLH068 are intron-less. The five intron-less genes are distributed across two clades, particularly clade nine (containing four intron-less genes) and clade five (containing the remaining one intron-less gene, VvbHLH091). The structural features of each grape bHLH gene are listed in Figure S2, while the others are listed in Figure S1. In *Salvia miltiorrhiza*, Zhang et al. (2015) also found intron-less genes mainly (four of six intron-less genes) distributed in one subfamily.

Exons with the same splicing phase at both ends are called symmetric exons, and an excess of symmetric exons and phase 0 introns are likely to facilitate exon shuffling, recombinational fusion, and protein domain exchange (Gilbert, 1987; Patthy, 1987; Zhang et al., 2015). According to the 464 exons analyzed herein, 202 exons are symmetric with phase 0 introns, two exons are symmetric with phase 1 introns, and 11 exons are symmetric with phase 2 introns. Among the 331 introns found in the bHLH genes, 276 are phase 0, 20 are phase 1, and 35 are phase 2. Therefore, our analysis of the bHLH gene structures in grapes strongly indicates a large diversity of bHLH transcription factors, which agrees with findings from *Salvia miltiorrhiza* (Zhang et al., 2015).

### Motif analysis

A total of 30 conserved motifs were characterized (motifs 1–30; Table S3). The grape bHLHs VvbHLH043 (in clade three), VvbHLH057 (in clade three), and VvbHLH021 (orphan) contain only two types of motifs, whereas the grape bHLH VvbHLH027 (orphan) contains the highest number of motifs (10 types). In *Salvia miltiorrhiza*, three types of motifs are repeated twice (Zhang et al., 2015), and in grapes, only motif two is repeated twice in VvbHLH012. Furthermore, certain conserved motifs are nested in specific clades. For example, motif 15 only exists in clade 10, motif eight only exists in clade 14, motif 14 only exists in clade 12, and motif 13 exists in clade one and clade 14. Moreover, every grape bHLH gene contains motif one and motif two. Motif 14 existed in many clades (clades 6, 7, 8, 13, and 15; Figure S3).

### Gene duplication and triplication analysis in the grape bHLH family

A previous study speculated that many bHLH genes may be generated by gene duplication in some plants (Sun et al., 2015). Therefore, we sought to detect duplicated gene pairs in the grape bHLH gene family. Forty-one duplicated gene pairs were found in grape genomes, including 26 high-stringency standard duplicated gene pairs and 15 low-stringency standard duplicated gene pairs (Table S4). Intraspecies synteny analysis showed that the many duplicated gene pair blocks were collinear, such as VvbHLH049–VvbHLH061 and VvbHLH058–VvbHLH032, which indicated that these gene duplications may derive from chromosome segmental duplication or a large-scale duplication event. Additionally, VvbHLH053–VvbHLH056 may be derived from a tandem duplication because they are located on the same chromosome.

We identified nine triplicated gene groups in the grape bHLH gene family, such as the VvbHLH058–VvbHLH032–VvbHLH020 group. Additionally, VvbHLH058, VvbHLH032, and VvbHLH020 were collinear, showing that the triplication may derive from chromosome segmental triplication or a large-scale triplication event.

All the triplicated gene groups in the grape bHLH family underwent purifying selection (Table 1). Purifying selection may generate genes with conserved functions or pseudogenization (Zhang, 2003). Based upon our Nr and GO function annotations and motif analysis, we found that the genes may be functionally conserved within a triplicated gene group such as the VvbHLH093–VvbHLH076–VvbHLH082 group (ICE), VvbHLH060–VvbHLH074–VvbHLH006 (ICE), VvbHLH007–VvbHLH068–VvbHLH009 (myc). The motifs of one bHLH family member are also similar to the other members in the same triplicated gene group (Figure S3).

**Table 1:** Selection pressure of each grape bHLH duplication gene pairs in triplicate gene-group.

**Each Gene Pairs in Triplicate Gene-Group**	**Non-Synonymous/Synonymous**
**GROUP 1**	
VvbHLH025- VvbHLH040	ka/Ks = 0.1814
VvbHLH054- VvbHLH040	ka/Ks = 0.2561
**GROUP 2**	
VvbHLH058- VvbHLH020	ka/Ks = 0.1500
VvbHLH032- VvbHLH020	ka/Ks = 0.1488
**GROUP 3**	
VvbHLH063- VvbHLH001	ka/Ks = 0.1958
VvbHLH077- VvbHLH001	ka/Ks = 0.1533
**GROUP 4**	
VvbHLH093- VvbHLH082	ka/Ks = 0.2203
VvbHLH076- VvbHLH082	ka/Ks = 0.2293
**GROUP 5**	
VvbHLH060- VvbHLH006	ka/Ks = 0.1974
VvbHLH074- VvbHLH006	ka/Ks = 0.2618
**GROUP 6**	
VvbHLH068- VvbHLH007	ka/Ks = 0.0497
VvbHLH009- VvbHLH007	ka/Ks = 0.0113
**GROUP 7**	
VvbHLH088- VvbHLH090	ka/Ks = 0.2519
VvbHLH052- VvbHLH090	ka/Ks = 0.2819
**GROUP 8**	
VvbHLH053- VvbHLH039	ka/Ks = 0.2762
VvbHLH056- VvbHLH039	ka/Ks = 0.4034
**GROUP 9**	
VvbHLH072- VvbHLH070	ka/Ks = 0.1978
VvbHLH071- VvbHLH070	ka/Ks = 0.2605

### Expression profile of grape bHLH genes

**Figure 2:**
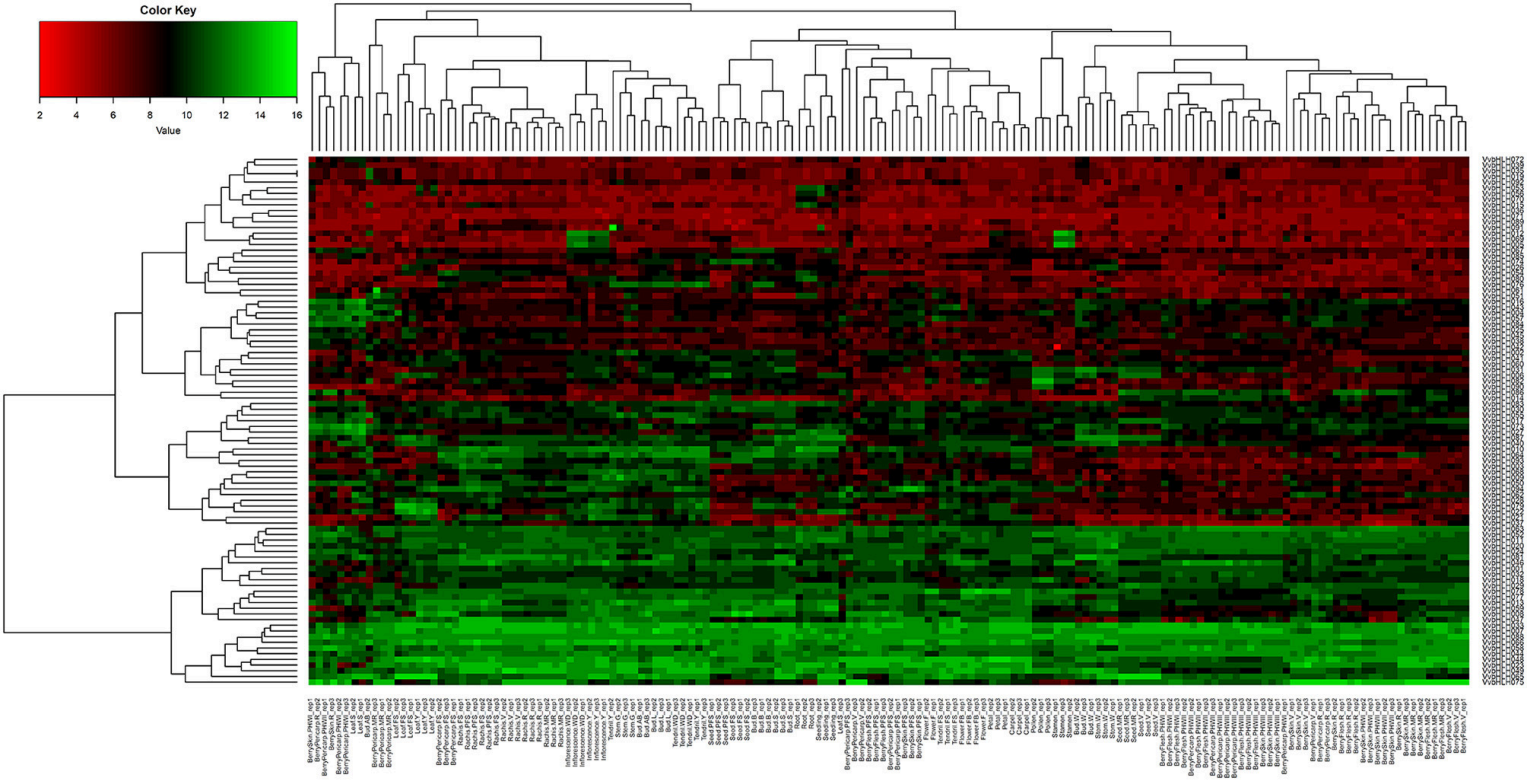
Heatmaps representing the expression profiles of grape bHLH genes in 49 tissues The log2 (RMA-normalized signal intensities value of grape bHLH genes) were used to represent the expression level. Details of tissue abbreviation name are listed in Table S5. “rep” represent the “replicate”.

The expression profiles of 94 grape bHLHs in 49 tissues (tissue name details are listed in Table S5) were analyzed based on the microarray expression profile data collected from GSE36128 on the NCBI database (https://www.ncbi.nlm.nih.gov/gquery/?term=GSE36128) (Fasoli et al., 2012). We found all 94 grape bHLHs expressed in the 49 tissues (**Figure 4**). The log2 values (RMA-normalized signal intensities value of grape bHLH genes) were used to represent the expression level. The log2 values vary from four to 15. Some genes were found to have a log2 value of more than 14 in a certain tissue; therefore, they were considered to be highly expressed in that tissue. VvbHLH007 (GO function annotation: response to abscisic acid stimulus, chitin, and insects; jasmonic acid mediated signaling pathway) was highly expressed in stamens, post-fruit set berry pericarp, latent bud, mid-ripening berry flesh, ripening berry flesh, post-harvest withering I (1st month) berry flesh, post-harvest withering II (2nd month) berry flesh, post-harvest withering III (3rd month) berry flesh, carpels, petals, post-harvest withering II (2nd month) berry pericarp, fruit set rachis, post-fruit set rachis, véraison rachis, mid-ripening rachis, ripening rachis, post-harvest withering II (2nd month) berry skin, green stem, well developed tendril, and mature tendril (**Figure 4**; Table S4). A list of tissue abbreviations can be found in Table S4 (Fasoli et al., 2012). VvbHLH005 (GO function annotation: pollen development; anther wall tapetum cell differentiation) was only highly expressed in stamens (**Figure 4**; Table S4), indicating that VvbHLH005 may play an important role in pollen development in grape stamens. VvbHLH048 (Nr annotation: pif4; GO function annotation: red or far-red light signaling pathway; regulation of biological quality) was highly expressed in post-fruit set berry skin, véraison berry skin, mid-ripening berry skin, young leaf, mature leaf, fruit set berry pericarp, post-fruit set berry pericarp, véraison berry pericarp, and post-fruit set berry flesh, showing that it may be involved in quantitative berry and leaf traits. VvbHLH075 was highly expressed in mid-ripening berry flesh and ripening berry flesh. VvbHLH088 was highly expressed in ripening berry flesh. VvbHLH048 was highly expressed in mid-ripening berry flesh and mid-ripening berry skin (Figure 2; Table S5). This indicates that they may play an important role in fruit ripening.

### Grape bHLH gene codon usage bias analysis

To understand whether grape bHLH genes have codon bias, the frequency of optimal codons (FOP), GC content, GC content on the 3rd site of a synonymous codon (GC3s content), the relative synonymous codon usage (RSCU), codon adaptation index (CAI), and codon bias index (CBI) were calculated. The average FOP was 0.397892 ± 0.040998, the average CAI was 0.197194 ± 0.023114, and the average CBI was −0.04456 ± 0.071419. The GC content for the 94 grape bHLH genes varied from 41.5 to 56.5% and the average GC content was 47.1%. The GC3s content for grape bHLH genes varied from 31.6 to 63.4% and the average was 45.29%. To further understand the influences of codon bias on gene properties, a correlation analysis was performed. GC content was positively correlated with CBI and FOP (*P* < 0.01), but is not strongly correlated with CAI; GC3s content is positively correlated with CBI and FOP (*P* < 0.01), but is not correlated with CAI (*P* < 0.01). Gene length and exon length are not strongly correlated with CBI, CAI, or FOP (Table 2).

**Table 2:** Correlation analysis.

	**FOP**	**CBI**	**CAI**
GC content	0.5^*^	0.5^*^	0.2^*^
GC3s content	0.6^*^	0.6^*^	0.2^*^
Gene length	0	0	0
Exon length	0	0.1^*^	0.1^*^

* *p < 0.01*.

The ENC in grape bHLH genes ranged from 30.28 to 60.96, with an average of 53.25. Among the 94 grape bHLH genes, only one gene (VvbHLH091) exhibited high codon bias (ENC < 35), indicating that in general, grape bHLH genes reflect random codon usage without strong codon bias (Li et al., 2016). Codon usage bias could be affected by many stressors including mutation pressure and natural selection (Li, 1987). If codon usage bias is corrected with GC content, we could deduce that the codon usage bias was affected by mutation pressure during its evolutionary history (Knight et al., 2001; Chen et al., 2004). The FOP and CBI of grape bHLH genes are positively correlated with GC and GC3s content, indicating that grape bHLH genes mainly evolved by mutation pressure.

**Figure 3:**
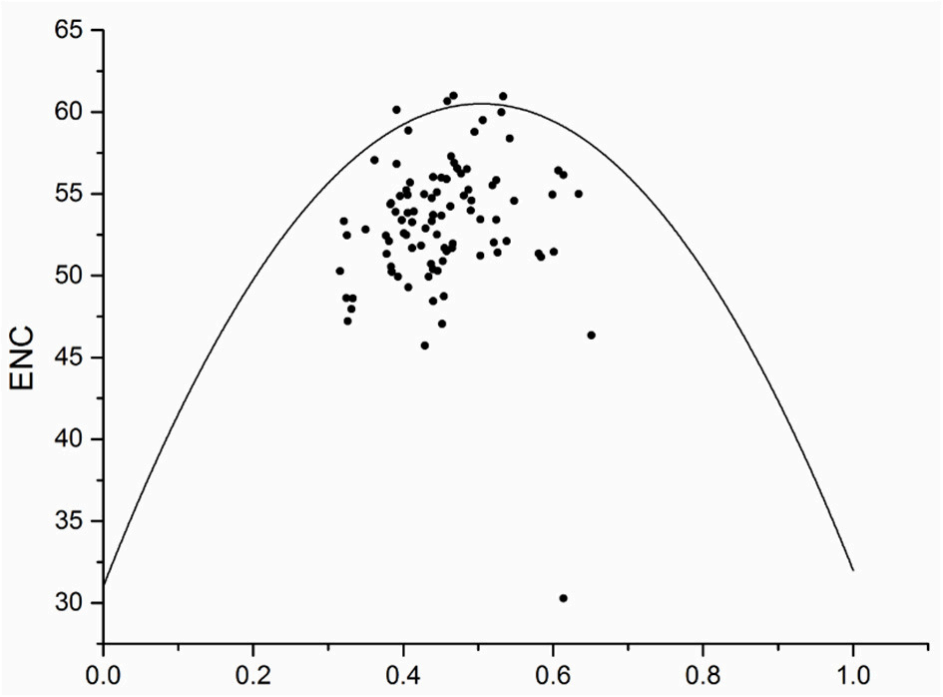
Effective number of codons ENC-plot showing relationship between ENC and GC3s.

An ENC plot was generated to explore the influence of GC3s on codon bias in grape bHLH genes. If a gene is located on the expected curve, the codons of that gene are not biased (Zhang et al., 2007). Here, all ENC values were lower or higher than expected and were located above or below the curve (Figure 3), indicating that other factors combined with mutation pressure affect codons. The distribution width of the GC3s might be related to the variation in the strength of directional selection against mutation pressure (Kawabe and Miyashita, 2003). In grape bHLH genes, the distribution of GC3s was between 0.314 and 0.651, indicating that grape bHLH genes evolved by mutation pressure.

**Figure 4:**
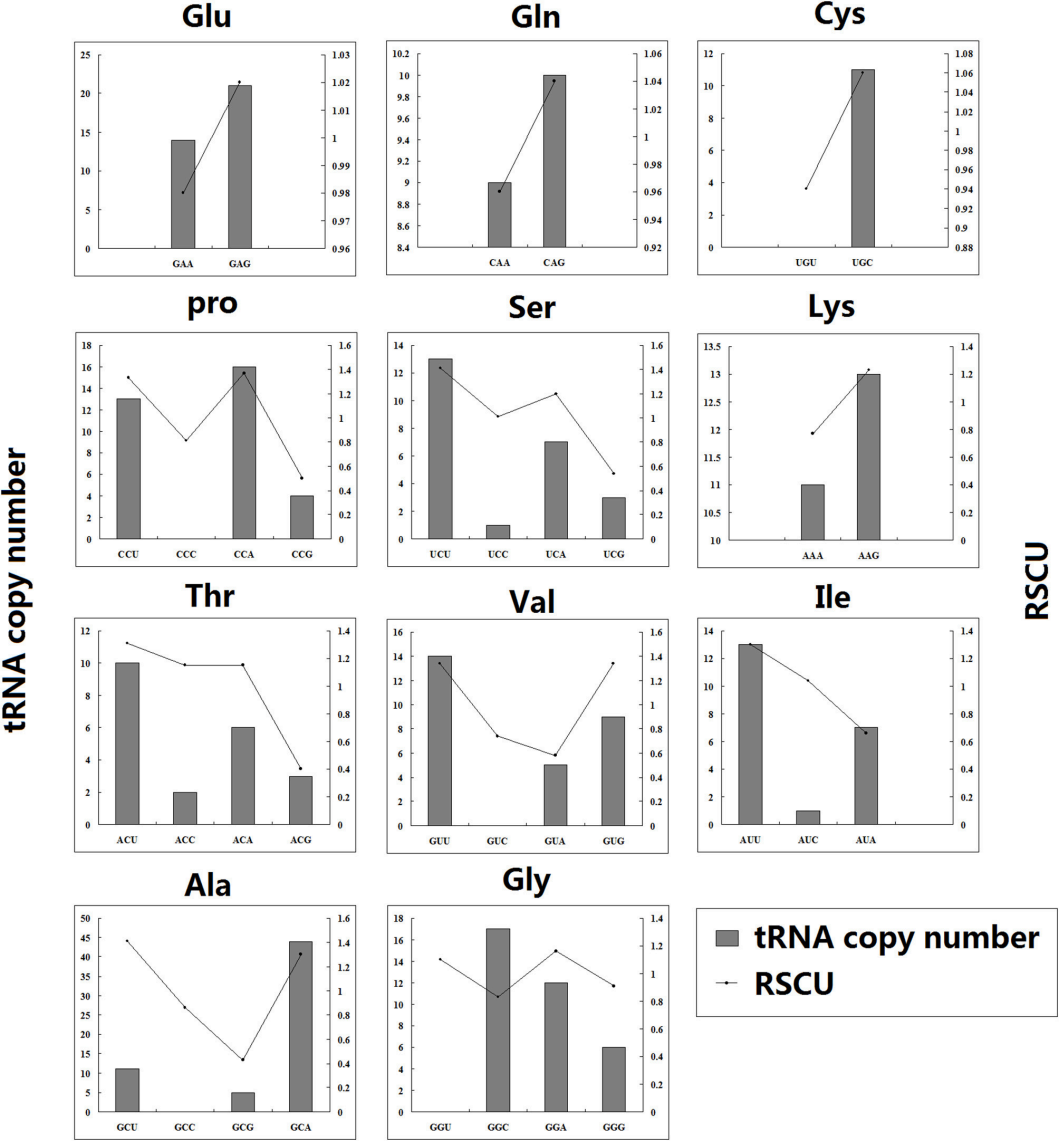
Relative synonymous codon usage in grape bHLH genes. tRNA copy number is shown on the left y-axis (gray bars) and RSCU (Relative Synonymous Codon Usage) is on the right y-axis (lines).

In grape bHLH genes, many codons with larger RSCU values had larger corresponding tRNA copy numbers, such as the synonymous codons in glu, gln, cys, thr, val, leu, pro, ser, and lys (Figure 4).

### The expression level of grape bHLH genes in abiotic stress tolerance

Based on the microarray expression profile data of the PEG treatment library and the control library (GSE31594; https://www.ncbi.nlm.nih.gov/geo/query/acc.cgi?acc=GSE31594), we found three bHLH genes were up-regulated 1.5-fold under a 1 h treatment with PEG. Four bHLH genes were up-regulated 1.5-fold under a 4 h PEG treatment. Sixteen bHLH genes were up-regulated 1.5-fold under an 8 h treatment with PEG (Figure S4; Table S6).

In total, 22 grape bHLH genes could be induced by PEG treatment. Among these 22 genes, 12 promoters contained ABRE elements (ABA responsive), 17 contained MBS or MER elements (Myb transcription factor binding; drought inducible), 15 contained HSE elements (Hsf transcript factor binding), and 17 contained G-box elements (bHLH transcript factor binding; Table S7).

**Figure 5:**
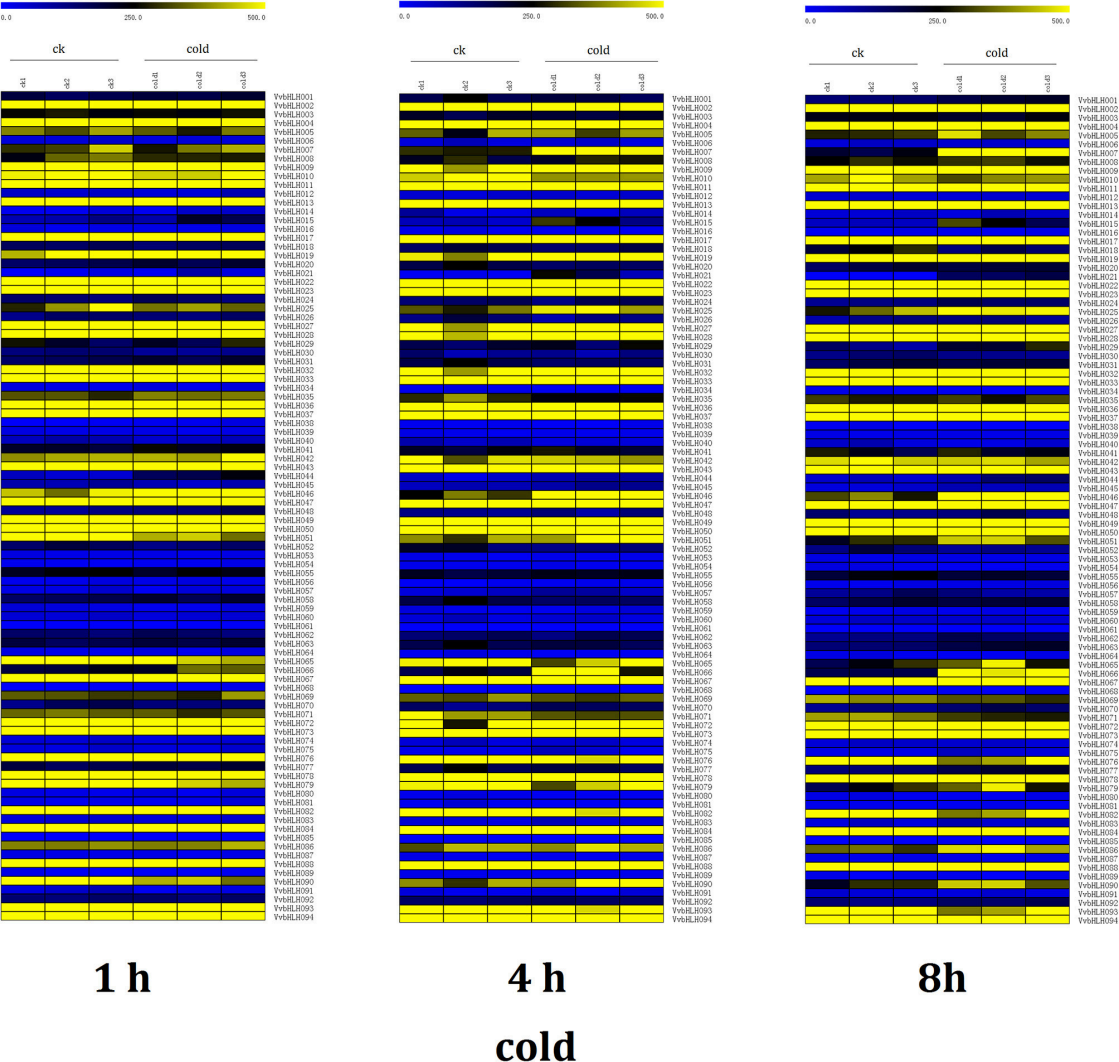
Heatmaps representing the expression profiles of grape bHLH genes under cold treatment RMA-normalized signal intensities value of grape bHLH genes were used to represent the expression level.

Based on the microarray expression profile data of the cold stress library and control library (GSE31594; https://www.ncbi.nlm.nih.gov/geo/query/acc.cgi?acc=GSE31594), we found seven bHLH genes were up-regulated 1.5-fold under 1 h of cold treatment (Figure 5; Table 3). Five bHLH genes were up-regulated 1.5-fold under 4 h of cold treatment. Thirteen bHLH genes were up-regulated 1.5-fold under 8 h of cold treatment (Figure 5; Table 3). In total, 17 grape bHLH genes could be induced by cold stress treatment including a homolog of MYC2 VvbHLH007 (Figure 5; Table 3). Among these 17 genes, four promoters contained ABRE elements, four contained LTR elements (low temperature responsive), 10 contained MBS or MER elements, 10 contained HSE elements, and 11 contained G-box elements (Table S7).

**Table 3:** Difference expression gene between ck and cold treatment.

	**Value of ck**			**ck‘**	**Value of cold treatment**			**Cold‘**	**Fold**	***P*-value**
**Gene name**	**ck1**	**ck2**	**ck3**		**cold1**	**cold2**	**cold3**			
**DEG BETWEEN CK AND COLD TREATMENT 1H**										
VvbHLH021	2.10	14.28	26.61	14.33	11.89	86.70	29.95	42.85	2.99	0.16
VvbHLH039	0.73	3.78	8.47	4.33	12.17	15.49	24.80	17.49	4.04	0.01
VvbHLH045	62.18	42.87	44.08	49.71	162.72	176.20	239.47	192.80	3.88	0.02
VvbHLH047	445.19	361.14	489.53	431.95	618.86	1, 104.60	831.11	851.52	1.97	0.07
VvbHLH061	1.27	1.11	2.64	1.67	0.69	1.57	5.86	2.71	1.62	0.23
VvbHLH066	187.19	192.55	228.94	202.89	214.10	361.45	342.72	306.09	1.51	0.06
VvbHLH068	1.93	2.05	4.95	2.98	9.26	2.56	16.76	9.53	3.20	0.09
**DEG BETWEEN CK AND COLD TREATMENT 4H**										
VvbHLH021	9.10	17.13	8.83	11.69	256.28	174.36	66.27	165.64	7.51	0.07
VvbHLH045	55.38	18.50	49.49	41.13	75.06	89.14	92.29	85.50	1.86	0.16
VvbHLH007	298.12	276.57	280.45	285.05	1, 175.30	925.11	494.27	864.89	1.76	0.05
VvbHLH047	249.06	375.78	305.60	310.15	1, 369.50	950.03	649.32	989.62	3.19	0.05
VvbHLH015	67.87	71.65	47.21	62.24	305.80	252.29	119.51	225.87	2.53	0.04
**DEG BETWEEN CK AND COLD TREATMENT 1H**										
VvbHLH021	3.91	3.85	1.39	3.05	158.36	155.58	181.99	165.31	54.16	0.00
VvbHLH083	32.09	59.85	32.27	41.40	80.60	45.14	66.48	64.07	1.55	0.18
VvbHLH046	28.12	31.90	48.93	36.32	72.78	60.65	70.58	68.00	1.87	0.02
VvbHLH090	217.47	296.32	293.81	269.20	450.02	458.91	332.50	413.81	1.54	0.06
VvbHLH052	217.47	296.32	293.81	269.20	450.02	458.91	332.50	413.81	1.54	0.06
VvbHLH047	321.52	387.41	271.73	326.89	1, 086.20	857.72	1, 041.80	995.24	3.04	0.01
VvbHLH065	167.85	238.48	299.32	235.22	346.41	490.64	268.64	368.56	1.57	0.13
VvbHLH079	167.85	238.48	299.32	235.22	346.41	490.64	268.64	368.56	1.57	0.13
VvbHLH066	171.62	177.90	179.35	176.29	561.44	480.46	539.36	527.09	2.99	0.00
VvbHLH068	3.14	8.76	9.52	7.14	10.97	9.32	11.98	10.76	1.51	0.12
VvbHLH015	58.75	65.04	58.58	60.79	323.91	251.12	170.73	248.59	4.09	0.03
VvbHLH011	1, 558.90	1, 958.30	1, 886.10	1, 801.10	3, 256.80	2, 740.20	3, 764.40	3, 253.80	1.81	0.03
VvbHLH029	155.24	117.41	84.02	118.89	205.26	203.01	288.18	232.15	1.95	0.07

*ck‘, average value of gene expression in ck; cold‘, average value of gene expression in cold treatment grape; DEG, different expression gene; fold, cold‘/ck‘*.

VvbHLH039 (org2), VvbHLH021 (bHLH92), and VvbHLH007 (MYC2) could be significantly induced by both PEG and cold treatment. The promoter of VvbHLH039 contained G-box elements, VvbHLH021 contained G-Box, LTR, and MBS elements, and VvbHLH007 contained ABRE, G-Box, HSE, and MBS elements. MBS elements could be responsive to drought and ABRE could be responsive to ABA. ABA-dependent pathways play a crucial role in the response of a plant to abiotic (drought, salinity, cold, and hypoxia) and biotic stressors. ABA-dependent transcription factors could be involved in cold, drought, and salinity stress gene expression such as MYC (Huang et al., 2012). The promoter of VvbHLH007 contained ABRE elements, so VvbHLH007 may be affected by ABA-dependent pathways and could be up-regulated by cold and drought stress. VvbHLH021 could be up-regulated both by PEG and cold stress, which may be due to the promoter of VvbHLH021 containing LTR elements and MBS elements. VvbHLH039 contained G-box elements, so it may be regulated by other grape bHLHs under cold or PEG treatments (Table S7).

Overexpression of CBF4 in Arabidopsis resulted in constitutive expressions of CRT/DRE containing stress responsive genes and enhanced tolerance to drought and freezing stressors (Haake et al., 2002; Chinnusamy et al., 2003). CBF could recognize the cold and dehydration responsive element CRT/DRE (Yang et al., 2005). Based on the microarray expression profile data of the CBF4 over-expression transgenic grape library (GSE29948; https://www.ncbi.nlm.nih.gov/geo/query/acc.cgi?acc=GSE29948), we found seven bHLH genes that were differentially expressed between a CBF4 over-expression transgenic grape compared with the control (Figure S5; Table S8). The promoter of VvbHLH037 and VvbHLH073 contained the CRT/DRE (A/GCCGAC) element (Table S7).

### The role of grape bHLHS in flavonoid and anthocyanin biosynthesis pathways

Previous studies have identified that the grape bHLH genes VvMYCA1 (ABM92332; VIT_215s0046g025601; Matus et al., 2010) and VvMYC1 (EU447172; VIT_207s0104g000902; Nr annotation in this study: TT8; Hichri et al., 2010) are involved in flavonol biosynthesis. Based on our GO or Nr function annotations, we found three additional genes that are potentially related to anthocyanin or flavonol biosynthesis including VvbHLH003 (GO annotation: anthocyanin-containing compound biosynthetic process; Nr annotation: transcription factor bhlh30), VvbHLH007 (GO annotation: positive regulation of flavonoid biosynthetic process; Nr annotation: MYC2), and VvbHLH010 (Nr annotation: myc-like anthocyanin regulatory protein; Table S2). Two of these three bHLH genes also have other functions: VvbHLH003 also functions in cellular response to phosphate starvation and VvbHLH007 functions in a jasmonic acid mediated signaling pathway, regulation of defense response to insects, response to chitin, response to abscisic acid stimuli, and response to desiccation. Therefore, these bHLHs genes are also involved in response to abiotic or biotic stimuli.

In our study, the promoters of the three bHLH genes contained HSE elements (Table S9), suggesting that they could be regulated by other Hsfs. Their promoters also contained MYB binding sites (MBS/MER) which are involved in drought response and G-box elements (Table S9), indicating that they could be regulated by MYB and bHLH transcription factors. The promoters of VvbHLH003 and VvbHLH007 contained ABRE elements, which are involved in ABA-dependent or independent stress tolerance (Chen et al., 2012).

### The majority of promoters of genes involved in flavonoid and anthocyanin biosynthetic pathways present G-Box/E-Box elements

Recently, Malacarne et al. (2015) and Costantini et al. (2015) identified genes with a role in the regulation of anthocyanin and flavonol content and composition including 64 genes involved in flavonoid biosynthesis, such as Myb domain protein 12 VIT_07s0005g01210 (which is involved in the regulation of flavonol biosynthesis), VIT_11s0052g01600 (UDP-glucose flavonoid 3-O-glucosyltransferase), VIT_11s0052g01630 (UDP-glucose flavonoid 3-O-glucosyltransferase), and VIT_16s0098g00860 (flavanone 3-hydroxylase). 60 of these gene promoters contained G-box or E-box elements (Table S8). Forty-four genes are involved in anthocyanin biosynthesis such as VIT_02s0033g00370 (VvMYBA4), VIT_02s0033g00380 (VvMYBA2), VIT_02s0033g00390 (VvMYBA2), VIT_02s0033g00410 (VvMYBA1), VIT_02s0033g00430, VIT_02s0033g00440, VIT_02s0033g00450 (VvMYBA3), VIT_08s0007g03560 (Anthocyanin membrane protein 1), and VIT_06s0009g02830 (flavonoid 3′, 5′-hydroxylase). 40 of these gene promoters contained G-box or E-box elements (Table S10).

## Discussion

### Expansion of the grape bHLH gene family may be drive by gene duplication

The basic helix-loop-helix (bHLH) transcription factor family is very large in eukaryotes (Ledent and Vervoort, 2001; Toledo-Ortiz et al., 2003) and many bHLH genes (100–170) have been identified in land plants (Carretero-Paulet et al., 2010). For example, 162 bHLH genes have been identified in Arabidopsis (Bailey et al., 2003), 167 bHLH genes in rice (Li et al., 2006), and 152 bHLH genes in tomato (Wang et al., 2015). In our study, we identified 94 bHLH genes in *V. vinifera*. The gene number of the grape bHLH gene family is large. Previous studies in tomato have shown that 103 genes were generated by gene duplication (Sun et al., 2015), indicating that the expansion of the tomato bHLH gene family is mainly driven by duplication. In *V. vinifera*, we found 31 duplicated gene pairs, showing that the expansion of the grape bHLH gene family may also be driven by duplication. A collinearity analysis showed that many duplicated gene-pair blocks were collinear and only one tandem duplication was found. Therefore, gene duplications of the grape bHLH gene family may mainly derive from chromosome segmental duplication or a large-scale duplication event. Both A whole genome replication event (γ event) and whole genome duplication event (WGD) could lead to chromosome segmental duplication or a large-scale duplication event. *Vitis vinifera* is thought to have undergone an ancient whole genome replication event (γ event), but not a WGD event (Jaillon et al., 2007). Therefore, the duplication in the grape bHLH gene family may mainly derive from the γ event. The γ event could have led to the triplicated arrangement in plant genomes and the generation of the triplicated gene group (Jaillon et al., 2007). In *V. vinifera*, we identified triplicated bHLH gene groups. Triplicated gene groups can be also found in many plants such as in Arabidopsis (Guo et al., 2017).

Both rice and Arabidopsis have undergone γ events and at least two WGD events, and grapes are believed to have only undergone a γ event (Jaillon et al., 2007). This may be the reason that grape contained fewer bHLH genes (94) compared with Arabidopsis (162) and rice (167).

### The functional divergence in the bHLH family and conserved functions in triplicated gene groups

The phylogenetic analysis showed that the grape bHLH gene family contained 15 clades (Figure 1). A previous study has shown that the Arabidopsis bHLH gene family contained 15 clades and some orphans (Feller et al., 2011), rice contained 22 clades (Li et al., 2006), and tomato contained 26 clades (Wang et al., 2015). The phylogenetic analysis showed that 17 grape bHLHs were tightly grouped with the AtbHLHs (Figure S1). A previous study showed that 43 tomato bHLHs were tightly grouped with the AtbHLHs. This indicates that the number of orthologous pairs between grape and Arabidopsis or between tomato and Arabidopsis is not large compared with the number of the bHLH gene family members. Different numbers of clades in different species and few orthologous pairs between different species showed that interspecific divergence existed in plant bHLH gene families.

Our Nr-annotation showed that the grape bHLH family contained some homologous bHLHs previously reported in Arabidopsis. However, the annotation did not contain the homologs of many bHLHs in Arabidopsis. For example, the grape bHLH family does not contain the homologs of FIT (involved in the regulation of iron uptake), AIB (involved in ABA signaling), or RHD6 (involved in root hair formation) in Arabidopsis. This supports the view that interspecific functional divergence exists in plant bHLH gene families.

In the grape bHLH gene family, there are a total of 464 exons; 215 exons are symmetric. Among the 331 introns found in the bHLH genes, 276 are phase 0. An excess of symmetric exons and phase 0 introns are likely to facilitate exon shuffling, recombinational fusion, and protein domain exchange (Gilbert, 1987; Patthy, 1987; Zhang et al., 2015). Therefore, our analysis of the bHLH gene structures in grapes strongly indicates a large diversity of bHLH transcription factors, which was similarly found in *Salvia miltiorrhiza* (Zhang et al., 2015). Our GO annotation and Nr annotation of the grape bHLH family showed that the grape bHLH family may be involved in many functions related to plant development and other biological processes (Tables S1, S2). Different grape bHLH genes had different expression modes in grape, showing that they may play roles in different tissues or biological processes too (Figures 2, 5, Figures S4, S5). Intraspecific functional divergence exists in the grape bHLH family and the functional divergence may be related to the structures of bHLH genes. In other species, such as Arabidopsis (Feller et al., 2011) and rice (Li et al., 2006), many different functions were identified in their respective bHLH families. This is consistent with our findings for *V. vinifera*.

All of the triplicate gene groups in the grape bHLH family underwent purifying selection (Table 1). Purifying selection may generate genes with conserved functions or pseudogenization (Zhang, 2003). Based upon our Nr and GO function annotations and motif analysis, we found that the genes may be functionally conserved within a triplicated gene group. The motifs of one bHLH family member are also similar to the other members in the same triplicated gene group (Figure S3), indicating that function divergence was weak within triplicated bHLH gene groups in grape.

### RSCU values of codons were correlated to tRNA copy numbers in grape

In grape bHLH genes, many codons with larger RSCU values had larger corresponding tRNA copy numbers in grape, such as the synonymous codons in glu, gln, cys, thr, val, leu, pro, ser, and lys (Figure 4). The translation efficiency constrains codon choice; therefore, the frequency of codon usage is positively correlated with tRNA availability (Ikemura, 1981). The above result showed that the codon usage bias of some grape bHLH genes may be related to the translation efficiency.

### Grape bHLH genes may play important roles in abiotic stress tolerance

In our study, we found 22 grape bHLH genes could be induced by PEG treatment and 17 grape bHLH genes could be induced by cold stress treatment. A homolog of bHLH92 (VvbHLH021) and a homolog of the MYC protein (VvbHLH007) could be significantly induced by both PEG and cold treatment. A previous study showed bHLH92 were involved in abiotic stress tolerance (Jiang et al., 2009). MYC proteins are known to function as activators in one of the ABA-dependent regulatory systems (Abe et al., 2003; Valliyodan and Nguyen, 2006). Over-expression of AtMYC2 resulted in an ABA-hypersensitive phenotype and improved osmotic stress tolerance of transgenic plants (Shinozaki and Yamaguchi-Shinozaki, 2007; Gao et al., 2011). In the grape bHLH family, we found three MYC genes, but only VvbHLH007 (MYC2) could be significantly induced by PEG or cold treatment.

The CBF cold-responsive pathway is related to cold acclimation and plays a key role in the CBF family (Thomashow, 2001). ICEs, a bHLH member that induces the expression of CBF, could induce the CBF family when temperatures are cold (Zarka et al., 2003). ICE1 can bind to MYC recognition elements in the CBF3 promoter and is important for the expression of CBF3 during cold acclimation (Chinnusamy et al., 2003). ICE2 participates in the response to deep freezing through the cold acclimation-dependent pathway in *Arabidopsis thaliana* (Gagne et al., 2002; Smalle and Vierstra, 2004). In grape, we found six ICE genes, but none were up-regulated under the PEG or cold treatments.

In our study, most of the PEG or cold induced grape bHLH gene promoters contained HSE elements (Table S7), suggesting these genes could be regulated by Hsfs. Most grape bHLH gene promoters contained MYB binding sites, which are involved in drought response (Table S7). This indicated that they could be regulated by MYB transcription factors under drought stress. Most grape bHLH gene promoters contained G-box elements, indicating that they may be regulated by other grape bHLHs. Many grape bHLH gene promoters contained ABRE and DRE elements, which are involved in ABA-dependent or independent stress tolerance (Chen et al., 2012). Therefore, these genes may play important roles in gene regulation in response to different stresses in grape.

Additionally, we found seven bHLH genes that were differentially expressed between a CBF4 over-expression transgenic grape compared with the control (Figure S5; Table S7). The promoter of VvbHLH037 and VvbHLH073 contained the CRT/DRE (A/GCCGAC) element (Table S7), indicating they may be regulated by the CBF4 protein.

The above results indicated that some grape bHLH family members may be regulated by the transcription factors CBF, Myb, Hsf, or other bHLHs, which could be regulated by other transcript factors or induced by abiotic stress. Some transcriptome data showed that the expressions of transcript factor gene families will change in response to abiotic stressors (Rienth et al., 2014, 2016; Rocheta et al., 2014). Transcriptome data from the grape cv. Trincadeira showed the MYB4-like gene could be up-regulated under water deficit (Rocheta et al., 2016). Hsf could also respond to cold, salinity, and drought stress (Kotak et al., 2007; Swindell et al., 2007; Hu et al., 2015; Wang et al., 2017). Therefore, Myb, Hsf, bHLH, CBF, and other transcript factors may interact creating a coordinated network, and play roles in abiotic stress response. Some bHLHs were found to be related to abiotic stress response in this study and may play roles in abiotic stress response and tolerance.

### Grape bHLH genes may play important roles in flavonoid and anthocyanin biosynthesis pathways

Flavonols are an important class of bioactive compounds in almost all higher plants such as grapes. They are most abundant in the flowers and skins of grape berries (Hmamouchi et al., 1996), thus are relevant to viticulture and enology. Flavonols, including anthocyanins and condensed tannins, are produced by the flavonoid biosynthetic pathway, and they represent one of the most important classes among flavonoids in terms of concentration (Flamini et al., 2013). Anthocyanins are the largest class of flavonoids (Welch et al., 2008), and constitute one of the most important families of secondary metabolites. In response to UV light, cold, drought, and other abiotic stressors, anthocyanins have been shown to be synthesized as protective compounds, and can also function to attract pollinators (Costantini et al., 2015).

Here, we found 60 of 64 genes in flavonoid and 40 of 44 genes in anthocyanin biosynthesis pathways had promoters that contain G-box or E-box elements. bHLH family members could bind to E-box or G-box cis elements and regulate gene expression (Atchley et al., 1999; Massari and Murre, 2000; Toledo-Ortiz et al., 2003; Li et al., 2006). Therefore, most of these genes may be regulated by grape bHLH family members. Previous studies have shown that MYB and WD40 transcription factors could interact with bHLHs to be involved with the biosynthesis of anthocyanins (Mol et al., 1998; Nesi et al., 2000; Winkel-Shirley, 2001) and flavonols (Walker et al., 1999; Nesi et al., 2000; Baudry et al., 2004). Additinally, G-box is also a cis-acting regulatory element involved in light responsiveness (Giuliano et al., 1988). Light could affect the flavonoid and anthocyanin biosynthesis and the expression of related genes (Azuma et al., 2012; Costantini et al., 2015). The G-box element may be the connector between light and flavonoid and anthocyanin biosynthesis.

We found that some bHLH genes were highly expressed in mid-ripening berry flesh, ripening berry flesh or mid-ripening berry skin (VvbHLH075, VvbHLH088, and VvbHLH048; Figure 4. Therefore, they may play an important role in fruit ripening and in flavonoid and anthocyanin biosynthesis pathways. Additionally, we found three genes that are potentially related to anthocyanin or flavonol biosynthesis based on GO and Nr function annotation. These three genes were expressed moderately in mid-ripening berry flesh, ripening berry flesh or mid-ripening berry skin.

The promoters of VvbHLH075, VvbHLH088, VvbHLH048, and three potentially related genes (VvbHLH003, VvbHLH007, and VvbHLH10) contained cis-acting elements involved in gibberellin-responsiveness (TATC-box/P-box), HSE, MYB binding sites (MBS/MER), and G-box elements. This showed that they may be involved in the gibberellin signaling pathway, and could be regulated by MYBs, Hsfs, and other bHLHs transcription factors. The promoters of VvbHLH003 and VvbHLH007 contained ABRE element which are involved in ABA-dependent or independent stress tolerance (Chen et al., 2012; Table S9). Therefore, these genes could also play important roles in response to different stresses in grape. The three genes that are potentially related to anthocyanin or flavonol biosynthesis are also predicted to have other functions involved in response to abiotic stressors based on the GO and Nr function annotation. They may not only be related to anthocyanin or flavonol biosynthesis but also to stressor/stimuli such as VvbHLH007 (MYC2). A previous study has shown that abiotic stressors (such as cold or heat stress) may regulate anthocyanin or flavonoid biosynthesis-related genes (Azuma et al., 2012; Rienth et al., 2014). It is possible that these biotic or abiotic stressors are related to the anthocyanin or flavonol biosynthesis in *V. vinifera*, and we hypothesize that bHLH members may be a connector between stressor and secondary metabolite biosynthesis in grape. Future research should seek to understand the relationships between the anthocyanin and/or flavonoid biosynthesis pathways and abiotic or other stimuli. Further studies on anthocyanin and flavonoid biosynthesis pathways and their stimuli will positively impact viticulture and breeding.

## Conclusions

Genome-wide identification and comparison of grape bHLHs with other plant species revealed that grape bHLH gene family is a large family. Chromosome segmental duplication or a large-scale duplication event played important roles in the expansion of grape bHLH gene family. Our study provided evidence for the roles of grape bHLH family members in many biological processes including abiotic stress tolerance and secondary metabolite biosynthesis. These results are useful for better understanding the complexity of the gene family and the relation between stressor and secondary metabolite biosynthesis in grape.

## Author contributions

PW, YW, and FR designed the study, PW wrote the manuscript. YL and QZ finalized the figures and tables. LS, HG, XJ and XW carried out most of the experiment, data analysis, and wrote the method section of the manuscript.

### Conflict of interest statement

The authors declare that the research was conducted in the absence of any commercial or financial relationships that could be construed as a potential conflict of interest.

## Supplementary material

Figure S1 Neighbor-joining phylogenic analysis of grape and Arabidopsis bHLH family.

Figure S2 Gene structure of grape bHLH family members.

Figure S3 Motifs identified from grape bHLH family members.

Figure S4 Heatmaps representing the expression profiles of grape bHLH genes under PEG treatment RMA-normalized signal intensities value of grape bHLH genes were used to represent the expression level.

Figure S5 Heatmaps representing the expression profiles of grape bHLH genes in CBF4 overexpression grape RMA-normalized signal intensities value of grape bHLH genes were used to represent the expression level.

Table S1 Nr function annotation of grape bHLH genes.

Table S2 GO function annotation of grape bHLH genes.

Table S3 Conserved sequence of motifs from grape bHLH family members.

Table S4 Duplication gene-pairs of grape bHLH gene family.

Table S5 List of tissue abbreviations.

Table S6 Difference expression genes between ck and PEG treatment.

Table S7 Cis-regulation elements of difference expression genes between ck and PEG, cold treatment and CBF4 over-expression grape.

Table S8 Difference expression genes between ck and CBF4 overexpression transgenic grape.

Table S9 Cis-regulation elements of genes potentially related to anthocyanin or flavonol biosynthesis.

Table S10 Promoter sequences of genes are involved in flavonoid biosynthesis and anthocyanin biosynthesis.
