# Supplementary figures and images for: Genome-Wide Characterization of bHLH Genes in Grape and Analysis of their Potential Relevance to Abiotic Stress Tolerance and Secondary Metabolite Biosynthesis
Source: Front Plant Sci. 2018 Feb 1;9:64. doi: 10.3389/fpls.2018.00064 (PMC5799661; doi:10.3389/fpls.2018.00064)

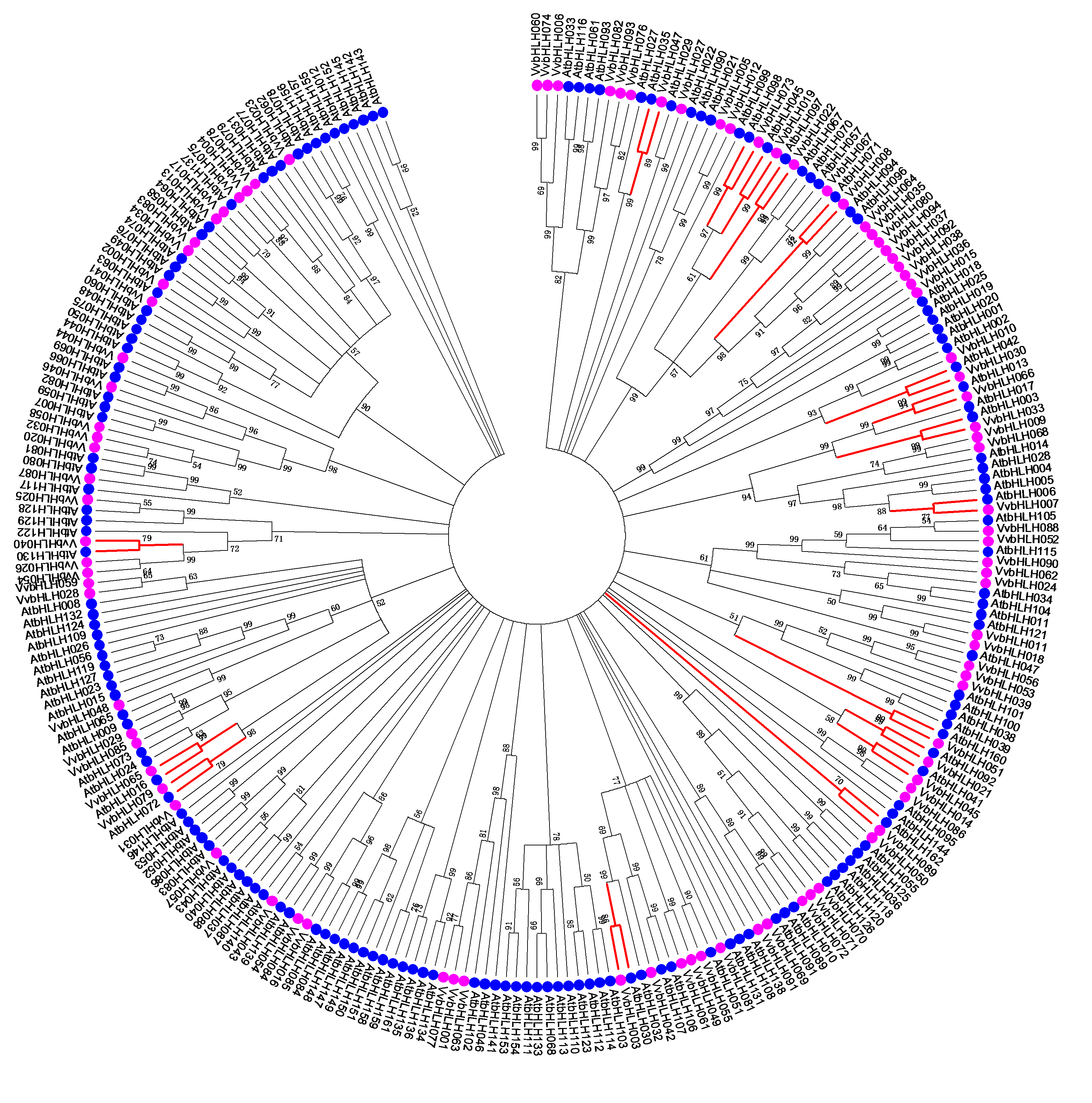

Supplement: Figure S1 — Neighbor-joining phylogenic analysis of grape and Arabidopsis bHLH family. [file Image1.TIF]

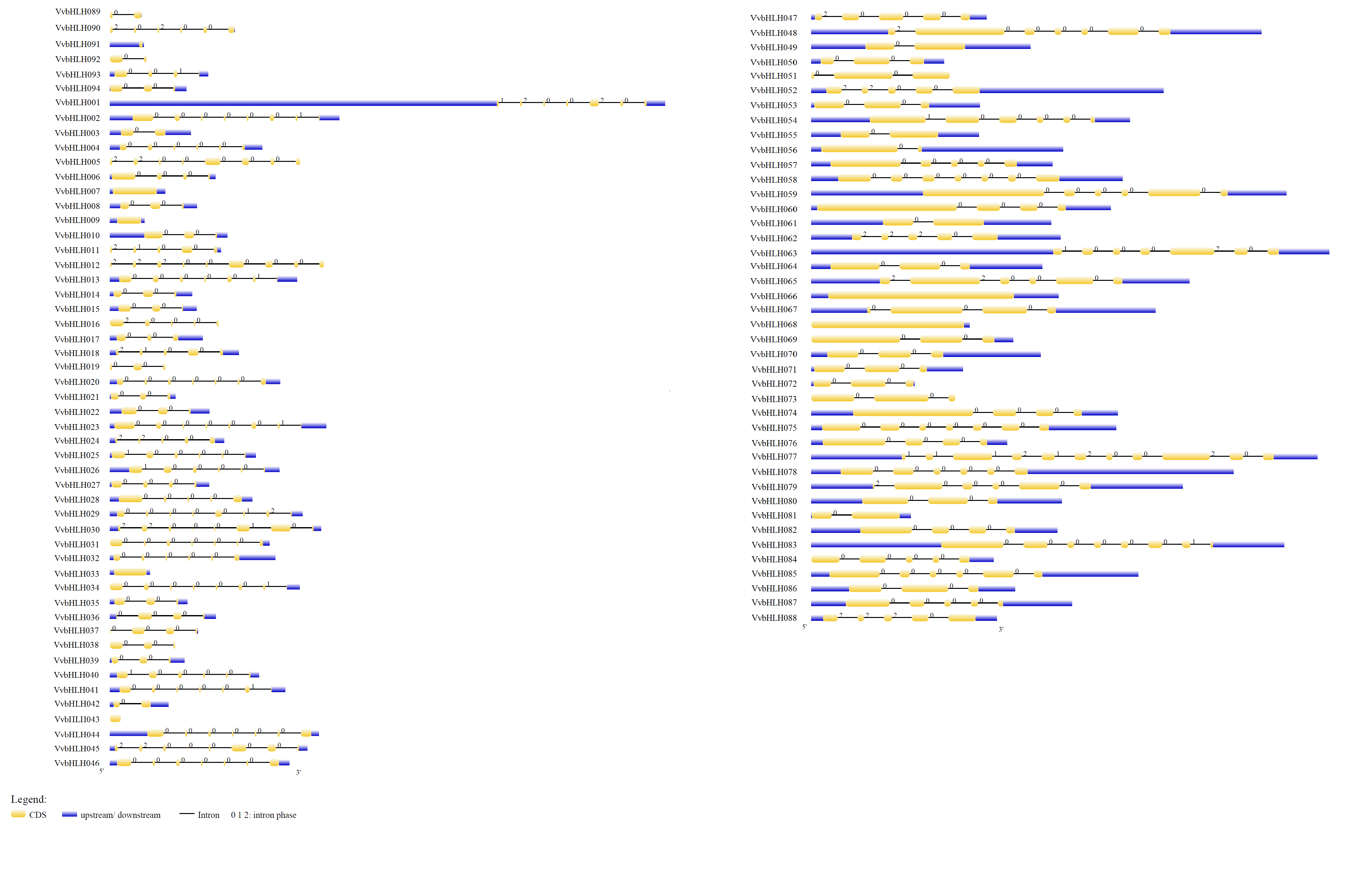

Supplement: Figure S2 — Gene structure of grape bHLH family members. [file Image2.TIF]

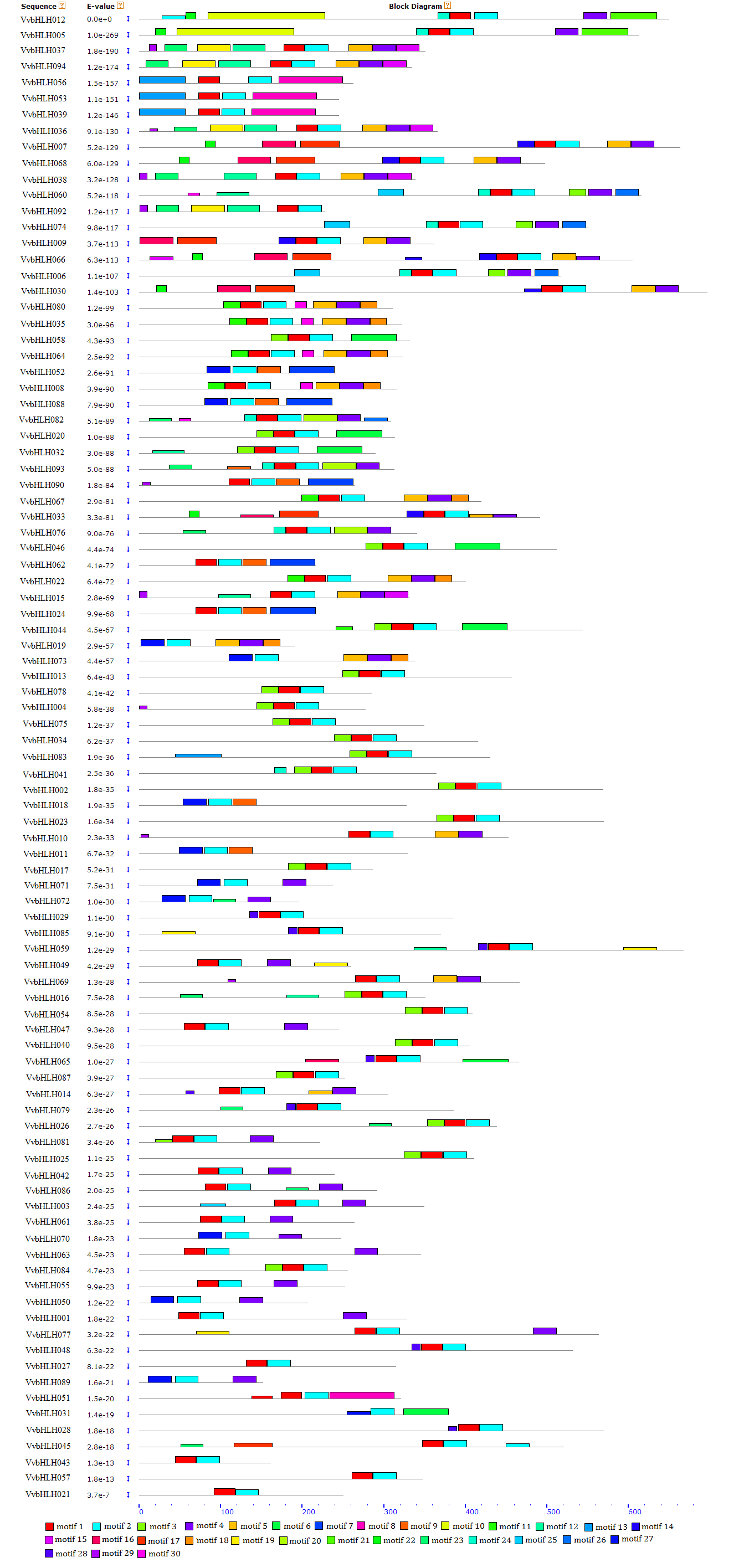

Supplement: Figure S3 — Motifs identified from grape bHLH family members. [file Image3.TIF]

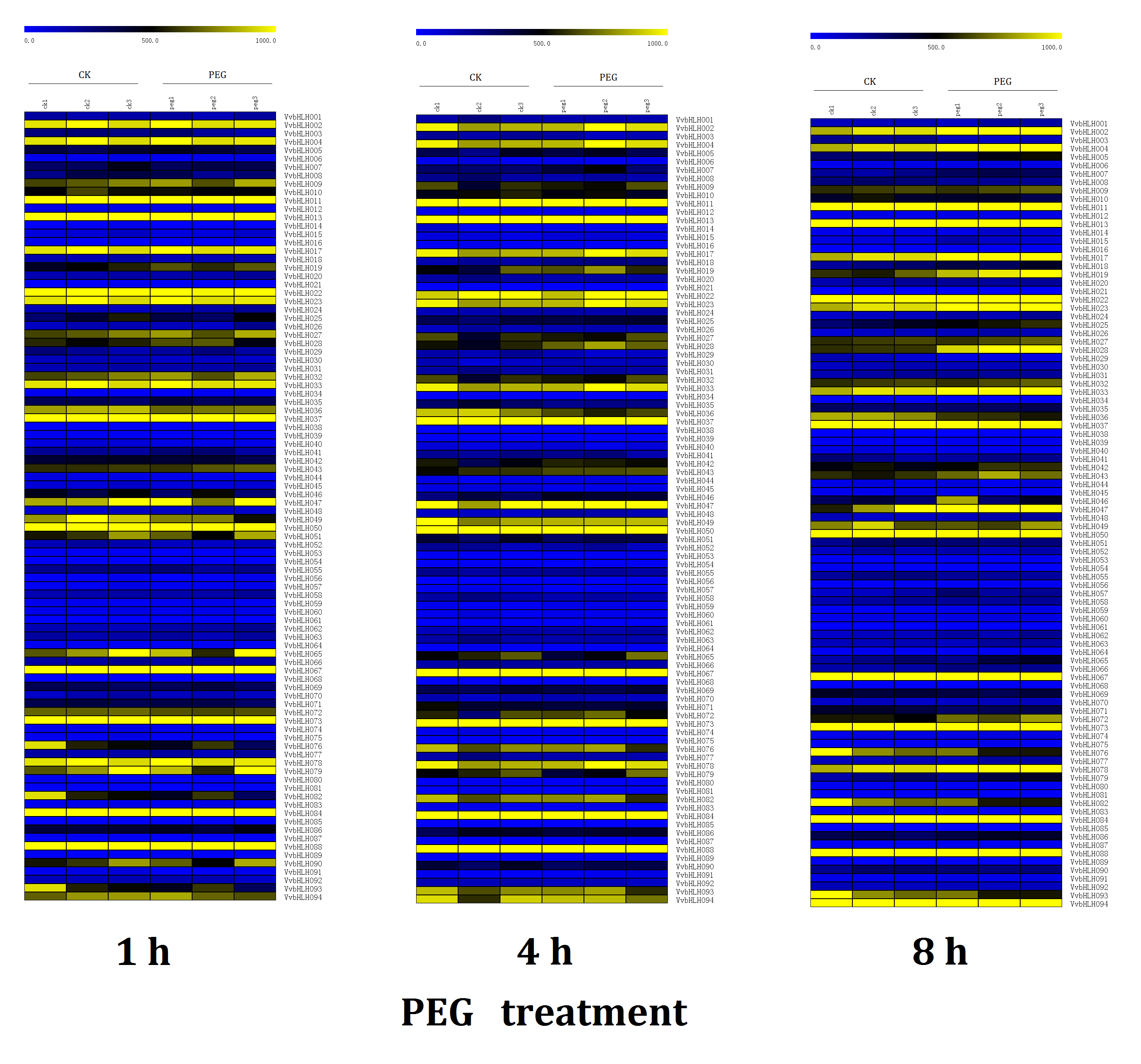

Supplement: Figure S4 — Heatmaps representing the expression profiles of grape bHLH genes under PEG treatment RMA-normalized signal intensities value of grape bHLH genes were used to represent the expression level. [file Image4.TIFF]

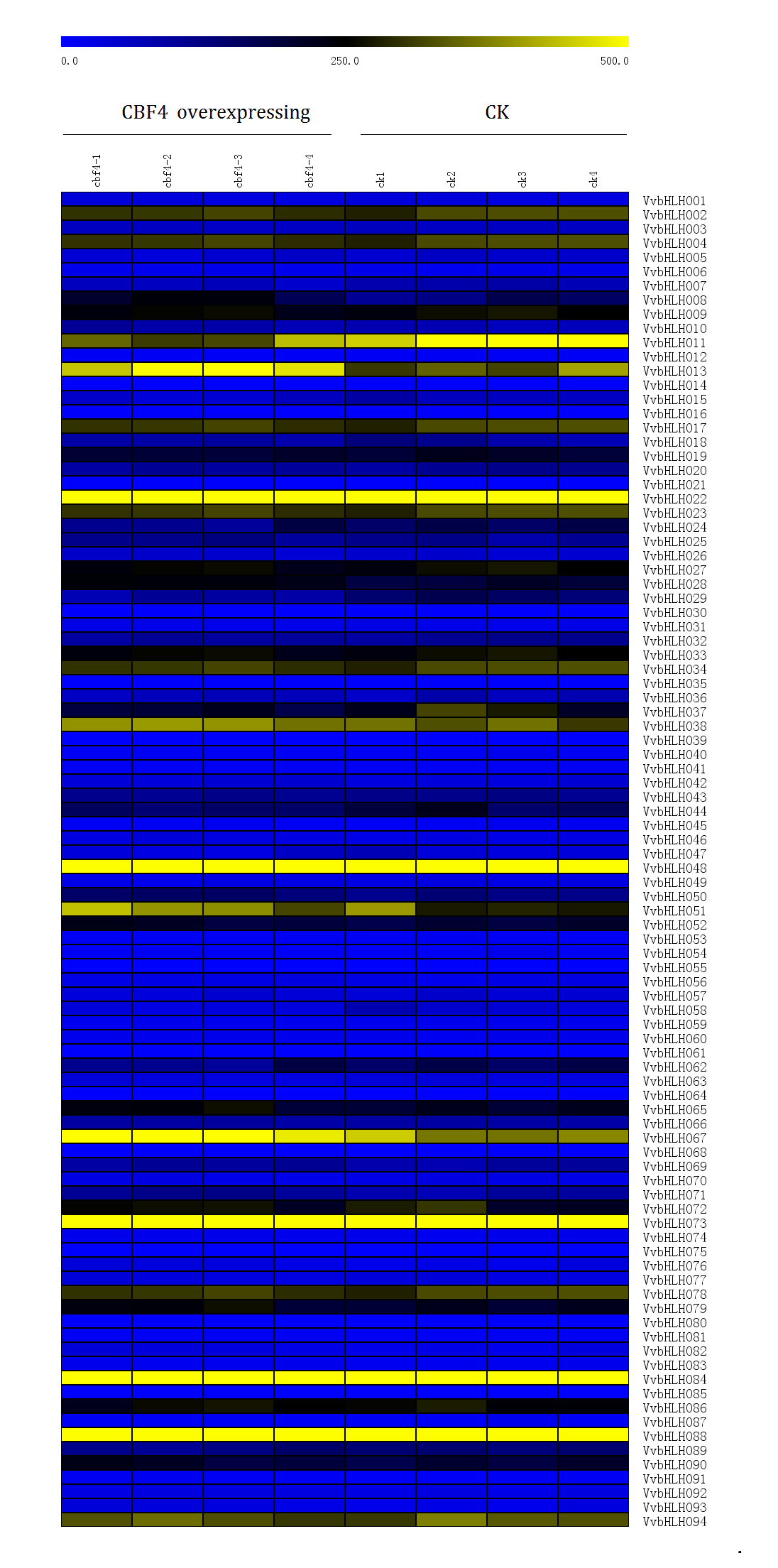

Supplement: Figure S5 — Heatmaps representing the expression profiles of grape bHLH genes in CBF4 overexpression grape RMA-normalized signal intensities value of grape bHLH genes were used to represent the expression level. [file Image5.TIFF]
